# The Role of the Stress Response in Metabolic Dysfunction-Associated Fatty Liver Disease: A Psychoneuroendocrineimmunology-Based Perspective

**DOI:** 10.3390/nu15030795

**Published:** 2023-02-03

**Authors:** Ilaria Demori, Elena Grasselli

**Affiliations:** Department of Earth, Environmental and Life Sciences (DISTAV), University of Genova, Corso Europa, 26, 16132 Genova, Italy

**Keywords:** liver steatosis, MAFLD, stress response, psychoneuroendocrineimmunology, cortisol, gut–liver axis, gut microbiota, inflammation, insulin resistance, eating behaviors

## Abstract

The novel term metabolic dysfunction-associated fatty liver disease (MAFLD), which has been proposed to describe the major cause of hepatic disease, pinpoints the coexistence of multiple metabolic disturbances and liver steatosis, giving rise to different phenotypic manifestations. Within the psychoneuroendocrineimmunological (PNEI) network that regulates body–mind interactions, the stress response plays a pervasive role by affecting metabolic, hormonal, immune, and behavioral balance. In this perspective, we focus on chronic psychosocial stress and high levels of cortisol to highlight their role in MAFLD pathogenesis and worsening. From a PNEI perspective, considering the stress response as a therapeutic target in MAFLD allows for simultaneously influencing multiple pathways in the development of MAFLD, including dysmetabolism, inflammation, feeding behaviors, gut–liver axis, and dysbiosis, with the hope of better outcomes.

## 1. Introduction

The accumulation of ectopic fat within hepatocytes is called liver steatosis. One cause of liver steatosis is alcohol abuse, known as alcoholic liver disease (ALD) [1]; however, it is estimated that a large proportion of the general population worldwide (about 25% on average) is affected by intrahepatic lipid accumulation, even in the absence of excessive alcohol consumption [2]. Until recently, this condition was referred to as non-alcoholic fatty liver disease (NAFLD) [3]. In NAFLD, the percentage of fat in the liver exceeds 5%, as determined by using biopsy as a diagnostic gold standard [4]. Fat accumulation is symptom-free and potentially reversible in its first stages [5], leading to possible diagnostic delays that can pave the way for more serious pathologies, encompassing steatohepatitis, fibrosis, cirrhosis, and eventually hepatocellular carcinoma [6]. Even more critical issues are posed on the treatment side, since an effective and safe pharmacological therapy has not yet been approved for NAFLD, whilst the strongest recommendations regard lifestyle modifications, particularly diet and physical activity, as being necessary to achieve optimal metabolic homeostasis [7]. However, thanks to the collection of new data on its etiopathogenesis, NAFLD has turned out to be a more complex condition than initially believed, to which genetic, metabolic, and environmental factors contribute simultaneously and/or in sequence [8]. It is therefore becoming increasingly clear that fatty liver disease must be treated as a systemic condition, which emerges from the relationships between the environment and the body’s regulatory systems. Psychoneuroendocrineimmunology (PNEI) is a scientific paradigm that highlights the bidirectional communications between the body’s regulatory systems, as well as their mutual involvement with the maintenance of the body’s health, or with transitions between physiological and pathological conditions [9]. PNEI integrates scientific knowledge from both psychological and biological sciences and explores the bidirectional connections between body and mind. A PNEI view is the basis of the science of integrated care and provides a common framework for the establishment of multidisciplinary teams for patient management [10].

Within the PNEI network, the stress response is at the crossroad of the reciprocal influences between the mind and the body, being triggered by both physical and psychosocial environmental challenges [11]. In this perspective, we want to highlight the pervasive role of the stress response in the pathogenesis and treatment of MAFLD, thus suggesting the targeting of psychosocial chronic stress for better and longer lasting therapeutic outcomes.

## 2. From NAFLD to MAFLD: One Letter Changes Everything

The acronym NAFLD has been used for many years to define intrahepatic fat accumulation not due to excess alcohol intake, and not related to other causes of liver damage [3]. In other words, the leading cause of liver disease worldwide has long been defined by what it is not, rather than what it is. In the last years, a panel of experts recognized the enormous progress made in understanding the mechanisms underlying the onset and worsening of NAFLD, and proposed a new nomenclature resulting in the replacement of one single letter in the acronym, which however radically changes the concept of the disease [12]. The novel umbrella term of metabolic dysfunction-associated fatty liver disease (MAFLD) highlights the coexistence of hepatic steatosis with an underlying state of systemic metabolic dysfunctions, also recognizing different phenotypic manifestations and multiple possible comorbidities [8].

MAFLD pathogenesis and worsening are complex and sustained by a variety of genetic, metabolic, and environmental factors with marked inter-individual variations in their effect size [12]. The initial “two hits” hypothesis suggested lipid accumulation as a first hit able to increase liver susceptibility to subsequent hits leading to inflammation and fibrogenesis [13], but this view is now regarded as an excessive simplification of a complex network of processes (“multiple hits”) that occur simultaneously and/or sequentially to determine different severities of the disease. Major hits include dietary and lifestyle factors, lipotoxicity, oxidative stress, inflammation, insulin resistance (IR), and intestinal dysbiosis [14,15]. The last two play essential roles and deserve further investigations, that have been carried out over the last decades. It is well known that in a state of IR and consequent hyperinsulinemia, adipose tissue lipolysis is not suppressed, de novo lipogenesis is enhanced, and mitochondrial β-oxidation is inhibited, resulting in increased delivery and accumulation of fat in hepatocytes [16,17]. On the other hand, the involvement of dysbiosis in MAFLD is a relatively new concept based on the existence of a gut–liver axis, which refers to the complex interactions between nutrition, gut microbiota, the intestinal barrier, and the liver. In the presence of dysbiosis and impaired functional integrity of the intestinal barrier, metabolic endotoxemia, Kupffer cell activation, and systemic low-grade inflammation occur, leading to MAFLD [18].

The reader will be able to find a plethora of literature on these mechanisms, even coming to understand some of the molecular pathways involved. Here, we want to highlight the pervasive role that the stress response plays in regulating these multiple hits when a PNEI perspective is used as a paradigm for approaching and treating MAFLD.

## 3. The Human Stress Response

The stress response is a highly adaptive allostatic mechanism that has evolved to enable the individual to cope with environmental challenges. It consists of a stereotypical systemic reaction involving the activation of neural, endocrine, and immune signals [19]. Two main axes can be identified: on the nervous side, the activation of the locus coeruleus/sympathetic nervous system and the release of catecholamines from the adrenal medulla into the bloodstream determine the “fight or flight response”, originally described by Walter Cannon [20]; on the hormonal side, the stressor stimuli activate the hypothalamus–pituitary–adrenal (HPA) axis, with the final release of glucocorticoids (GCs, cortisol in humans and corticosterone in rodents) [21]. Triggering of the stress system increases arousal, motor reflexes, attention, cognitive function, cardiovascular activity, blood pressure, and breathing rate. Metabolic effects encompass stimulation of glycogenolysis, gluconeogenesis, and lipolysis to allow the rapid mobilization of body energy from storage sites to heart, muscle, and brain [21]. On the immune side, catecholamines suppress antiviral responses whilst enhancing the transcription of proinflammatory genes. Cortisol exerts well-known anti-inflammatory actions, besides stimulating the type 2 immune response [22,23].

By responding to ancestral actual physical threats (i.e., predatory animals and hostile conspecifics), the stress response ensures short-term benefits, such as the capacity to fight or flight and to improve wound healing and recovery from injuries and infections. However, in the modern era, actual physical dangers have been displaced by chronic perceived psychosocial stressors. Psychosocial stress is induced by imagined or existing adverse social situations in our daily lives that we feel unable to cope with. Table 1 lists the Axis IV stress categories presented in the DSM-IV-TR [24], with examples of specific psychosocial stressors.

Chronic exposure to negative psychosocial factors is a major cause of hyper-activation of the stress response and chronic high levels of cortisol that become maladaptive and give rise to long-term costs in terms of enhanced systemic inflammation and higher vulnerability to viral infections and cancer [25]. By binding to ubiquitous glucocorticoid receptors (GRs), cortisol produces systemic allostatic adaptations in carbohydrate and lipid metabolism and in energy balance, also affecting the digestive system, brain and behaviors, and the immune system [26].

## 4. Stress, Cortisol and MAFLD

Fatty liver disease has long been considered the hepatic manifestation of metabolic syndrome (MetS) [27]. Given the widespread metabolic effects of GCs, the role of the stress response and HPA axis hyperactivation in the pathogenesis of MetS has been envisaged and investigated [28,29,30]. The literature evidence on the association between psychosocial stressors and MetS was recently reviewed in a meta-analysis by Kuo et al. [31], suggesting that chronic stress could be as harmful as other known cardiometabolic risk factors such as sedentary lifestyle.

Looking at MAFLD more specifically, several lines of evidence from cellular, animal, and clinical studies support the possible role of high cortisol levels and psychosocial stressors in MAFLD onset and worsening [32]. In rat hepatocytes, GCs alone or in combination with insulin promote gluconeogenesis, glycogenolysis, and lipogenesis, whilst decreasing VLDL secretion [32,33,34,35,36,37,38]. These results have been endorsed in animal models, since the administration of GCs to rodents stimulates hepatic lipid synthesis and decreases fat utilization [32,39,40,41]. Besides stress, hypercortisolism in humans can be studied in Cushing’s syndrome and in patients under GC therapy [42]. Cushing patients are prone to developing MetS, with central obesity, hyperglycemia, dyslipidemia, and IR; accordingly, case reports and studies outlined a NAFLD prevalence of 20% in a small group of Cushing patients [43], as well as the presence of NAFLD and the development of NASH in patients receiving high doses of GCs [44,45,46]. Targer et al. [47] suggested that mild chronic hyperactivity of the HPA axis leading to subclinical hypercortisolism was associated with the severity of NAFLD.

Related to metabolic dysregulation are the effects of GCs on eating behaviors. An increased caloric intake, particularly from carbohydrates and fats, results in obesity and MAFLD. GCs stimulate food intake through several pathways involving both homeostatic and hedonic mechanisms [48]. First, cortisol acts as a negative feedback on the secretion of CRH, thus relieving the anorexigenic signaling of the latter [49]. Second, cortisol stimulates the expression of the orexigenic peptides NPY and AgRP at the arcuate nucleus (ARC) of the hypothalamus [50] and the production of the hunger hormone ghrelin in the stomach [51]. Third, cortisol stimulates the intake of energy-dense “comfort food” which activates the dopamine reward circuitry [52]. Finally, GCs reduce the satiety signaling to the ARC by promoting leptin and insulin resistance [53,54].

Other stress-induced metabolic adaptations that can be related to MAFLD regard the inhibition of thyroid function. Prolonged activation of the stress response suppresses the hypothalamus–pituitary–thyroid (HPT) axis and the conversion of thyroxine to the biologically active triiodothyronine (T_3_) in peripheral target tissues [21,55]. Consequently, the expression of UnCoupling Protein-1 (UCP-1) in brown adipose tissue, which is normally stimulated by T3, is diminished and further down-regulated by GCs [56,57,58]. Altogether, these effects contribute to blunted metabolic rate and energy expenditure, which can promote MAFLD. Accordingly, hypothyroidism has been associated with MAFLD, and multiple experiments in vivo and in vitro demonstrated that iodothyronines are lipid-lowering agents able to prevent and reduce hepatic steatosis by affecting the expression of key genes of lipid homeostasis [59,60].

In addition to creating a metabolic environment suitable for MAFLD establishment, chronic stress shows the potential to trigger several hits of MAFLD pathogenesis. For instance, it also affects the digestive system and the gut microbiota, i.e., the gut–liver axis, which is involved in MAFLD. Stress and HPA activation can cause intestinal dysbiosis. In mice exposed to social disruption, the relative abundance of the genus Bacteroides was decreased, while that of Clostridium increased [61]. Dysbiosis and psychological stressors can also increase gut permeability, the so-called “leaky gut” [62,63], allowing bacteria, toxins, metabolites, and antigens to cross the epithelium and activate a mucosal immune response. Of note, a bottom-up communication is also present, as cytokines produced by activated immune cells can in turn enhance the response of the HPA axis [64]. Moreover, the germ-free animal model demonstrated the existence of gut microbiota signals which are essential for the development and functionality of the HPA axis [65,66]. The gut–liver axis also consists of the bidirectional pathways between the liver and the intestine regarding bile production, secretion, metabolism, and actions. Bile acids affect the function and composition of the gut microbiota, which in turn is responsible for bile acid metabolism [67,68]. Bile acids and their metabolites act as signaling molecules through their binding to target cell receptors, such as the farnesoid X receptor (FXR), which is expressed in intestinal and liver cells and involved in ALD and NAFLD. Therefore, dysbiosis-induced disruption of bile acid and FXR functions is another possible mechanism that links chronic stress and MAFLD [68].

Experimental and clinical evidence demonstrates the association between dysbiosis and MAFLD [69]. Fecal microbiota transplant from obese donors (both mice and humans) to germ-free mice can induce hepatic lipid accumulation [70,71]. Different dysbiotic types in NAFLD are associated with ethnic, age, and sex differences, in addition to the presence of other disorders such as obesity and MetS [70,72,73]. Moreover, the severity of NAFLD and its worsening is also associated with differences in the microbiota composition and metabolite production [69,74].

Last but not least, chronic stress is accompanied by an increase in the proinflammatory response [75]. The underlying mechanisms encompass the involvement of the gut–liver axis (dysbiosis, leaky gut) as described above, the complex immunomodulatory effects of GCs, and cortisol resistance [76]. The latter occurs when immune cells become less sensitive to the anti-inflammatory effects of GCs in order to compensate for their continuous secretion [77]. Different mechanisms underlying GC resistance have been proposed and documented, including modulation of GC availability (e.g., by influencing corticosteroid-binding globulin, the multidrug resistance (MDR) P-glycoprotein transporter, and 11β-hydroxysteroid dehydrogenase activity), as well as GR dysfunction due to reduced expression, binding affinity to its ligand, nuclear translocation, DNA binding, or interaction with other transcription factors [76]. The significance of GC resistance is not completely clear, but it has been suggested that under exposure to persistent social-environmental stressors and rejection, either actual or perceived, this phenomenon allows both high metabolic energy and high inflammatory cytokines to limit infections if an injury occurs [25,78]. Evidence of the association between social stress and inflammation has been obtained through the Trier social stress test (TSST) [79], in which participants are asked to give a speech and perform difficult mental arithmetic. When this task was performed in front of socially rejecting raters, participants exhibited a greater inflammatory profile and GC resistance, with respect to individuals who performed the TSST in the absence of such raters [80]. Inflammation can either precede or follow liver steatosis. In both cases, it predicts adverse prognostic outcomes such as fibrosis and steatohepatitis [81,82]. Activation of the NLRP3 inflammasome brings about liver inflammation and fibrosis, and it has been proposed to be implicated in NAFLD development and worsening [83].

Despite the evidence of adverse metabolic, neuro-immuno-endocrine, and behavioral effects of psychological stressors that can be associated with MAFLD, the direct role of the stress response in the development of the disease is largely unexplored and unclear. Considering preclinical models, an interesting work by Corona-Perez et al. [84] applied a restraint protocol to induce chronic stress in male rats. They recorded hepatic inflammation and fibrosis but not steatosis. However, hepatic lipid accumulation was present in restrained rats when they were fed a high-sucrose diet. In our opinion, these findings are interesting because they highlight the interplay between stress and diet, which is often underrated in clinical practice. On the other hand, in a mouse model of non-alcoholic steatohepatitis (NASH), chronic restrain stress even attenuated hepatic lipid accumulation by disrupting bile acid homeostasis. However, the anti-steatotic effect was ascribed to β-muricholic acid, a metabolite that is not synthesized in humans [85].

Several authors proposed the stress response and the dysregulation in the HPA axis are the links between NAFLD and psychiatric disorders (such as depression, bipolar disorders, or schizophrenia) in human beings, because of the multiple effects of stress on mood, behaviors, metabolism, and inflammation [86,87]. Particularly, long-term psychological stress is highly correlated with the onset of major depressive disorder (MDD), possibly via the central actions of up-regulated inflammatory cytokines [88]. It appears that a bidirectional association exists between MDD and NAFLD, since MDD mediates NAFLD occurrence and development, while NAFLD aggravates depressive states [87,89]. The same happens in children, since pediatric NAFLD is frequently accompanied by psychological distress, whilst children with psychiatric disorders are at greater risk of hepatic steatosis [90].

More direct data on the role of the stress response in MAFLD have been collected in two interesting studies carried out with participants from a Kangbuk Samsung Health Study in South Korea. The first is a longitudinal cohort study aimed at evaluating the association between NAFLD and autonomic imbalance, as estimated by using heart rate variability (HRV) [91]. The authors did not include stressful lifestyles in their model, but the results indicated that the risk of NAFLD was increased by low parasympathetic activity and recently increased sympathetic activity, which are clear features of activation of the stress response. This autonomic imbalance can cause IR, enhanced lipolysis, and increased inflammation [92,93,94].

The second study is cross-sectional, and it is the first one to investigate the association between perceived stress and the prevalence of NAFLD [95]. The results showed that higher perceived stress was independently associated with an increased prevalence of NAFLD, particularly in male and obese participants. These findings drive attention to consider the sex-related differences in the stress response, which could be important for different therapeutical approaches. In fact, men appear to have a higher sympatho-adrenal activation upon performance-related psychosocial stressors than women [96,97]. Regarding obese people, the authors assigned the strong association with the inflammatory environment to be associated with obesity [98].

**Figure 1:**
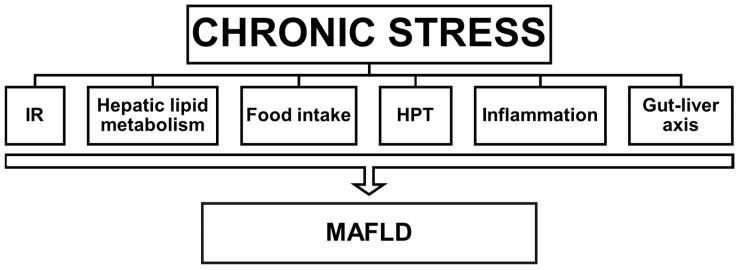
Major potential multiple roles of psychosocial chronic stress in MAFLD. Chronic activation of the stress response leads to high GC levels that exert multiple effects related to MAFLD development. GCs induce insulin resistance (IR) and affect hepatic lipid metabolism by increasing fat delivery from the adipose tissue to the liver, where de novo lipogenesis is stimulated whilst fat utilization and secretion are diminished. Chronic stress stimulates the intake of energy-dense food and decreases the functionality of the HPT axis, thus decreasing metabolic rate and energy expenditure. By means of different mechanisms that include GC resistance, chronic stress enhances inflammation, which is also related to induced gut dysbiosis and leaky gut (gut–liver axis).

To sum up, there are several mechanisms through which chronic psychosocial stress and consequently elevated cortisol levels can sustain the onset and worsening of MAFLD. Direct evidence has been collected in some cases, but further investigations are needed. Figure 1 depicts a schematization of the major potential multiple roles of chronic stress in MAFLD.

## 5. Considering Stress in the Anamnestic Process and Therapeutic Strategies for MAFLD Patients

So far, we tried to highlight the importance of psychosocial chronic stress in the development of MAFLD, because of the multiple PNEI effects elicited by the activation of the stress response, particularly the HPA axis. Research in PNEI demonstrates that cognitive and emotional functioning, under the influence of genetics and psychosocial factors, plays a fundamental role in orchestrating behavioral and biological stress responses, able to affect significantly not only the transitions between physiological and pathological conditions but also therapeutic outcomes [99].

We suggest using the scientific knowledge from PNEI as a common language among different health practitioners taking care of a MAFLD patient in a multidisciplinary team that includes a clinical psychologist and/or a psychiatrist able to evaluate the patient’s level of psychosocial stress.

Several tools are available to investigate an individual’s reactivity to stress and the presence of psychosocial stressors. While a measure of cortisol levels “per se” is not strictly indicative of chronic stress, such an analysis is significant when combined with other anamnestic data within a PNEI view. Cortisol levels can be measured in serum or urine, but, less invasively, the patient themself can collect salivary samples at different times of the day. Salivary cortisol levels reliably estimate serum cortisol because of high correlations between the two [100]. It is well known that cortisol production by the adrenal cortex follows a circadian rhythm, with the highest levels in the early morning and the lowest at night [19]. Morning cortisol levels mainly reflect chronic stress related to social and work environment [101,102], but repeated sampling could be of interest in order to design the complete circadian pattern related to the single patient’s lifestyle. Saliva represents a very complex biological fluid that can be used to accurately and non-invasively measure several PNEI mediators, including hormones, enzymes, antibodies, and cytokines, that can reach the saliva through diffusion, active transport, or ultrafiltration [103]. Therefore, salivary samples can be used to concomitantly assess different biological markers related to stress [104], including pro-inflammatory cytokines [105], and α-amylase, which is a correlate of sympathetic activity under a variety of conditions of physical and psychological stress [106,107]. Hair cortisol analysis is another simple, well-tolerated, and non-invasive procedure that requires only small amounts of hair to assess long-term integrated cortisol secretion, and is robust to acute situational influences [108].

On the other hand, the sympathetic activation within the stress response can be objectively quantified by heart rate variability (HRV), which is determined by the variation between heartbeats, known as RR intervals [109]. HRV is decreased when the heart rate increases by sympathetic activity; whereas, HRV is increased when the heart rate is reduced by parasympathetic activity [110].

Apart from these analyses, which may not always be applicable in a diagnostic clinical context, it is fundamental to assess the patient’s major life events, socioeconomic condition, sleep quality, and self-perceived stress through face-to-face interviews. This thorough anamnestic process can be further enhanced by using validated questionnaires. Health practitioners will find a wide variety of tools able to evaluate psychophysical symptoms related to chronic stress, such as anxiety, depression, poor sleep, fatigue, coping styles, and so on. As an example, the association between psychosocial stress and the risk of acute myocardial infarction was established in the INTERHEART study, in which the authors investigated the presence of psychosocial stressors using simple questionnaires that assessed family, occupational and economic problems, major life events in the past year, locus of control, and depression [111]. Confirming the validity of such an approach in MAFLD, a recent report showed that assessment of lifestyle factors helped to identify liver fibrosis in obese patients with NAFLD [112].

Once risk factors for chronic stress and/or high HPA axis activation with increased cortisol levels have been detected, stress management strategies should be recommended. As an example, mindfulness meditation is meant to cultivate “the awareness that emerges through paying attention on purpose, in the present moment, and nonjudgmentally to the unfolding of experience moment by moment” [113]. Mindfulness-based interventions have proven effective in stress-related symptomatology, eating behaviors, and inflammation [114,115,116], which can be beneficial in MAFLD. Similar to mindfulness, PNEI-based meditation (PNEIMED) aims to bring the Buddhist tradition closer to the Western world, by combining meditation sessions with the transmission of scientific knowledge from PNEI. PNEIMED can lower stress-induced cortisol levels and stress-related psychosomatic symptoms [117,118], thus its effectiveness on MAFLD would be worth testing. Some authors proposed the introduction of a psychologist within a multidisciplinary team caring for patients with MAFLD, in order to optimize the motivation and adherence to lifestyle modifications with the help of behavioral therapy [119,120]. The same is supported by Funuyet-Salas et al. [121] who reported that low perceived social support was associated with greater anxiety, depressive symptoms, and worse physical and mental health in NAFLD, contributing to a low probability of successful adherence to lifestyle modifications. We agree with and emphasize these views by bringing to attention that stress management interventions would simultaneously affect eating behaviors and diet adherence, as previously explained by the effects of stress on appetite, food choice, and metabolism [48,122,123,124,125,126,127]. Moreover, psychological support to MAFLD patients with a high risk of psychological distress could prevent worsening into full-blown psychiatric disorders such as MDD, which shows a bidirectional relationship with MAFLD as described above [89].

Diet is of the utmost importance for MAFLD prevention and therapy. In the PNEI view, nutritional interventions exert complex effects on body–mind interconnections, being able to affect metabolic regulation, mood, and behaviors, including stress reactivity and regulation of food intake. Particularly, interventions aiming to ameliorate intestinal inflammation, dysbiosis, and leaky gut would have the most widespread impact on the gut–liver axis and the microbiota–gut–brain axis [18,128,129,130,131]. The presence of gut dysbiosis, increased intestinal permeability, and inflammation should be routinely assessed in MAFLD patients with non-invasive methods. Urine samples can be used to evaluate the grade of dysbiosis according to the urinary levels of indican and skatole. In addition, urinary excretion of orally administered probe molecules (such as mannitol and lactulose), that might be absorbed via paracellular pathways, is a simple indirect measure of intestinal permeability. Alternatively, serum levels of markers of mucosal damage (such as zonulin or LPS) can be investigated [132].

As for prevention and therapy, dietary factors and supplements exert important action on intestinal barrier integrity. Gluten, fats, emulsifiers, bile acids, ethanol, and fructose display detrimental effects. Particularly, ethanol and fructose should be avoided in MAFLD, due to their overlapping dysregulatory actions on hepatic lipid metabolism [133]. On the other hand, fiber, short-chain fatty acids, glutamine, zinc, polyphenols, and vitamins A and D enhance barrier integrity by improving the mucus layer, tight junctions, and regulatory T cell functions [134]. Evidence is accumulating supporting the beneficial effects of probiotics in MAFLD through different routes. Probiotics may improve transaminase levels, hepatic lipid accumulation, NAFLD activity score, and inflammation [135,136]. VSL#3, a widely studied multistrain probiotic, protects intestinal barrier integrity in both adult and pediatric MAFLD patients [137,138]. Prevention of gut leakiness by intestinal microbiota modulation with probiotics also leads to an attenuated HPA response in rats [131]. Translational research in this field is still too limited, and further investigation on the combined use of probiotics and psychobiotics [139] is needed for broad-spectrum microbial action against MAFLD [140].

Increasing evidence points to the role of commensal organisms in early programming and later responsiveness of the stress system [131]. Upon restraint stress, germ-free mice show an exacerbated HPA activation, which is ameliorated by probiotic treatment in a time-dependent manner [141], meaning that early gut colonization is necessary for the normal development and activity of the stress response. The early disruption of the stress response can in turn lead to impaired signaling between the paraventricular nucleus of the hypothalamus and the feeding-related circuitry [126]. Moreover, eubiotic or dysbiotic gut microbes and their metabolites shape the immune system in early life, resulting in healthy or pathological imprinting, respectively [142]. Altogether, these observations pinpoint the role of perinatal programming of disease development in adult life, a hypothesis that has been increasingly validated since the pioneering work of Barker, who found epidemiological links between small birth size and greater cardiovascular and metabolic risks later in life [143,144]. Both maternal obesity and prenatal exposure to GCs (the latter can occur following hyperactivation of the maternal stress response) have programming effects on hepatic lipid metabolism, and increase susceptibility to NAFLD in the offspring [145].

**Figure 2:**
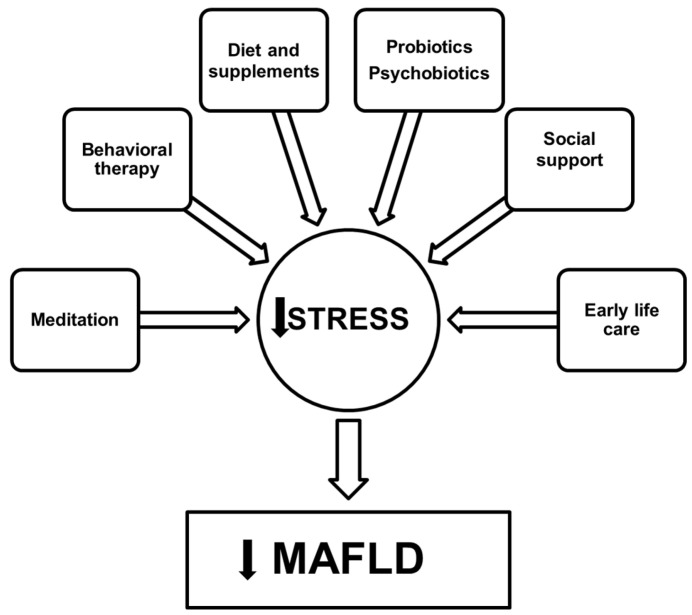
Major suggested interventions for stress management with potential beneficial effects on MAFLD. Meditation interventions have proven effective on cortisol levels, stress-related symptomatology, eating behaviors, and inflammation. Behavioral therapy can help the patients with motivation and adherence to lifestyle modifications, including diet, and appropriate psychotherapy is mandatory for patients at high risk of psychiatric comorbidities such as MDD. Diet remains the main pillar in MAFLD therapy, with particular emphasis on its anti-anti-inflammatory and microbiota-regulating properties, which can be enhanced with appropriate supplements, probiotics, and psychobiotics, thus affecting the gut–liver and microbiota–gut–brain axes. Social support and accurate health care should be reserved for pregnant mothers and newborns, given the role of perinatal programming of disease development in adult life, including MAFLD.

In addition to prenatal stress, children and adolescents may experience a wide range of psychosocial stressors listed in Table 1, including problems with family and social environment (family discord, sexual or physical abuse, loneliness, neglect, bullying), educational problems, homelessness, and poverty. Furthermore, the recent COVID-19 pandemic has caused a dramatic increase in psychological distress in childhood and adolescence [146]. When combined with the increasing prevalence of MAFLD among children [147], all these observations should draw the attention of national health services and governments to the mental and physical health of pregnant mothers and newborns, in order to avoid a global burden of interconnected psychophysical pathologies, including stress-related disorders, dysmetabolism, and MAFLD. During the gestational period, pregnant mothers mainly consult with gynecologists, who should collaborate with stress and nutrition experts to ensure the health of the microbiota–gut–brain axis of the mother and child from early pregnancy and beyond. Figure 2 depicts the major suggested interventions for stress management with potential beneficial effects on MAFLD.

## 6. Limitations

The stress concept is a difficult one when it comes to being analyzed as a risk factor in research or in clinical settings. It is a complex variable that can depend on ethnic, cultural, age, and sex differences. Moreover, since the work of Lazarus [148], it has become evident that the individual’s stress response depends on the balance between the stressful stimulus and the ability to cope with it. In each person, genetic and environmental factors (mainly life experiences), as well as personality traits are all involved in the perception of an event as more or less stressful and in its symbolic meaning. Therefore, caution should be exercised when analyzing self-reported stress; these data should be combined with more objective markers and constructs inferred from validated questionnaires.

In regard to an individual’s stress responsiveness and coping ability, the metabolic outcomes of the stress response can differ significantly. For example, it has been postulated that stress habituation, which develops a lower stress reactivity, might result in different patterns of fat distribution and metabolic trade-offs compared to non-habituators, who exhibit higher cortisol levels, visceral fat accumulation, and increased cardiovascular risk [149]. Similarly, sex-related differences in metabolic regulation, particularly glucose homeostasis and energy balance, should be considered [150]. Epidemiological data from different countries reported a higher prevalence of MetS in women than in men, as well as a female predisposition to central adiposity [151]. Concerning the stress response, data from rodents and humans established a greater reactivity of the HPA axis in females than males [152]. In addition, it has been reported that when faced with chronic stress, women seem more prone to unhealthy lifestyles, including physical inactivity and eating disorders [153,154]. Despite all of this, a recent meta-analysis found that women have a lower risk of NAFLD than men, but also a higher risk of progression towards fibrosis [155].

As previously described, the measure of cortisol levels should be considered together with other markers and constructs, since high cortisol levels and stress are not always mutually related. Moreover, even in the absence of an overactive HPA axis, increased sensitivity of adipose tissue to GCs may cause visceral obesity and IR, leading to the development of MetS, and potentially MAFLD, in patients without high cortisol levels [29,156,157]. Furthermore, the paradoxical phenomenon of hypocortisolism has been described in patients with post-traumatic stress disorder and in some stress-related pain disorders such as fibromyalgia, low back pain, and chronic pelvic pain [158]. The triad of hypocortisolemic symptoms includes pain, fatigue, and stress sensitivity and can develop after a prolonged period of stress and HPA axis hyperactivity. The consequent hyporesponsiveness of the HPA axis may be interpreted as an adaptation that may also have beneficial effects on the organism, but the concept is still a matter of speculation and debate [158,159].

Indeed, research on the link between stress and MAFLD is still in its infancy and there is a strong need for well-designed animal and human studies, especially longitudinal studies to examine long-term behavioral and metabolic effects under conditions of chronic stress. The lack of specific studies makes our perspective still descriptive and speculative. However, based on the existing literature, we are convinced that framing the experimental and epidemiological data within a PNEI view would help to unravel the role of stress in the onset and development of MAFLD.

## 7. Conclusions

In the PNEI paradigm, the separation between psychological and physical pathologies is not possible, and the stress response is a crucial hub of body–mind interconnections able to affect metabolism, immunity, and behaviors. The view of MAFLD as a disorder of the PNEI network can help pursue new diagnostic and therapeutic approaches for better outcomes. Modulation of the gut microbiota and bioactive metabolites can be achieved by both nutritional and psychological tailored interventions with repercussions on the entire PNEI network, including the gut–liver axis, the gut–brain axis, the inflammatory response, and energy homeostasis. Stress and diet reciprocally influence each other, and both share the potential to ameliorate the silent MAFLD epidemic worldwide [160].

## Figures and Tables

**Table 1:** Psychosocial stressors according to the DSM-IV-TR.

Category	Psychosocial Stressor	Examples
1	Problems with primary support group	Family discord, child and marital issues, conflict with parents, death in the family, sexual or physical abuse, child apprehended by child services
2	Problems related to the social environment	Socially isolated, no friends, living alone, no social supports, manipulated by peers, challenges with peer groups
3	Educational problems	Academic problems, stress from education program
4	Occupational problems	Unemployed, stress at work, workplace disputes
5	Housing problems	Homelessness, underhoused, no fixed address, stress due to housing situation
6	Economic problems	Financial difficulties, poverty, issues with disability support payments
7	Problems with access to health care	Health care provider not accessible, no health provider, no health insurance
8	Problems related to interaction with the legal system/crime	Legal charges, court appearance, recent release from jail, probation
9	Other psychosocial and environmental problems	Any issues related to immigration that were not social or occupational in nature, including refugee status and refugee claimant process

## Data Availability

Not applicable.
